# KnotResolver: tracking self-intersecting filaments in microscopy using directed graphs

**DOI:** 10.1093/bioinformatics/btae538

**Published:** 2024-09-03

**Authors:** Dhruv Khatri, Shivani A Yadav, Chaitanya A Athale

**Affiliations:** Division of Biology, Indian Institute of Science Education and Research Pune (IISER Pune), Pashan, Pune, Maharashtra 411008, India; Division of Biology, Indian Institute of Science Education and Research Pune (IISER Pune), Pashan, Pune, Maharashtra 411008, India; Division of Biology, Indian Institute of Science Education and Research Pune (IISER Pune), Pashan, Pune, Maharashtra 411008, India

## Abstract

**Motivation:**

Quantification of microscopy time series of *in vitro* reconstituted motor-driven microtubule transport in “gliding assays” is typically performed using computational object tracking tools. However, these are limited to non-intersecting and rod-like filaments.

**Results:**

Here, we describe a novel computational image-analysis pipeline, KnotResolver, to track image time series of highly curved self-intersecting looped filaments (knots) by resolving cross-overs. The code integrates filament segmentation and cross-over or “knot” identification based on directed graph representation, where nodes represent cross-overs and edges represent the path connecting them. The graphs are mapped back to contours and the distance to a reference minimized. The accuracy of contour detection is sub-pixel with a robustness to noise. We demonstrate the utility of KnotResolver by automatically quantifying “flagella-like” curvature dynamics and wave-like oscillations of clamped microtubules in a “gliding assay.”

**Availability and implementation:**

The MATLAB-based source code is released as OpenSource and is available at https://github.com/CyCelsLab/MTKnotResolver.

## 1 Introduction

Microtubules (MTs) are an essential component of the cell cytoskeleton, and their interaction with molecular motors serves to generate forces required for organelle positioning and the assembly of supra-molecular structures like the mitotic spindles, cilia, and flagella (Woolley 2000, Guérin *et al.* 2010). The collective properties of MT growth and interactions result in complex networks *in vivo*. However, quantifying the mechanical properties in such an intracellular environment has proven to be difficult due to the crowded nature of the intracellular space (Ellis 2001). As a result, the collective mechanical properties of both MTs and motors have been extensively studied *in vitro*. One prominent method used to examine collective transport by molecular motors is by immobilizing motors on the surface and allowing filaments to bind to them, which results in transport of MTs, referred to as “gliding assays” (Howard *et al.* 1989, Nitzsche *et al.* 2010, Sumino *et al.* 2012). Typically in such MT “gliding assays” with short filaments, i.e. of lengths 5 μm or less, are observed almost as rod-like objects in microscopy in the presence of either kinesin (Scharrel *et al.* 2014) or dynein (Monzon *et al.* 2018, Jain *et al.* 2019). Actin filament motility driven by myosin, however, shows highly curved and bent structures at similar length scales (Kron and Spudich 1986, Ishijima *et al.* 1991, Kron *et al.* 1992). This is consistent with the reported persistence length of MTs *in vitro* at thermal equilibrium of the scale of millimetres, while actin has an average persistence length of the order of ∼10 μm (Gittes *et al.* 1993). Additionally, highly curved filaments are seen in “gliding assays” either due to torsional forces exerted by myosin resulting in filaments “twirling” (Beausang *et al.* 2008). Such buckled MTs have also been experimentally observed when filaments were bent using optical tweezers in order to measure their rigidity (Kurachi *et al.* 1995). Molecular motor-driven filament “gliding assays” modified such that one end was clamped also resulted in highly curved filaments, the dynamics of which were tracked manually (Gittes *et al.* 1996). We have demonstrated a comparable end-clamping of MTs in a dynein “gliding assay” results in wave-like motion of filaments, that self-intersect (Yadav *et al.* 2024). Such self-intersections proved difficult to track with existing tools. Axonemes reconstituted using a bottom-up approach have also been observed to form complex curved structures, which are challenging to track using conventional filament tracking tools (Guido *et al.* 2022). Thus, tracking curved and self-intersecting filaments, i.e. knots, from time series are of wider relevance for quantification of collective motor-driven filament transport studies.

Quantifying such “gliding assays” typically involves detecting and tracking the movement of individual filaments in microscopy time series. Some of the widely used tools include a MATLAB-based tool, FIESTA, which was shown to track MTs with sub-micron precision (Ruhnow *et al.* 2011), an active contour-based approach applied to track actin filaments, JFilament that works as a FIJI plugin (Li *et al.* 2009, Smith *et al.* 2010) and MTrack, a versatile FIJI plugin (Kapoor *et al.* 2019). While these tools are optimized for a general “gliding assay,” they assume non-intersecting filaments that are straight over the time series, assumptions that are violated in either complex geometries or crowded environments.

Multiple algorithmic approaches to resolving networks from microscopy have been developed, including a general algorithm for the decomposition of filamentous networks (DeFiNe) by weighted, undirected graph representation, and roughness minimization that can resolve simulated, cellular, and astronomical images of meshes of filaments (Breuer and Nikoloski 2015). At a whole cell scale, NeuriteTracer has been used to automatically detect neuronal extensions based on low-level segmentation and skeletonization (Pool *et al.* 2008), which has been integrated with a graph theoretical representation in simple neurite tracer (Arshadi *et al.* 2021). At an organismal scale, images of *Caenorhabditis elegans* worms at high density were resolved by combining skeletonization with a graph-theoretical representation of worm contours as edges and adjacencies as nodes (Raviv *et al.* 2010). Representing networks as open curves using B-spline surface representation of filaments optimized by gradient-descent to the image, combined with image-formation model to detect and segment unknown numbers of filaments (Xiao *et al.* 2016). Thus, across problem-types, the application of graph representations has been useful to resolve filaments in complex geometries. However, these have, to our knowledge, not yet been applied to single filaments with cross-overs in combination with tracking.

In this study, we describe a novel approach to segment and track single MT filaments in time-series data that undergo self-intersections or knots, which we refer to as KnotResolver. Our approach combines active contours seeded by an initial guess based on an intensity threshold and geometry, the representation of the intersecting segments as elements of a directed graph, and distance minimization between time-frames by comparing curves predicted by the directed graph with an initial reference that is not intersecting. We demonstrate the utility of our approach by applying it to resolve knots in both *in silico* and *in vitro* oscillations of clamped MTs described in a recent study (Yadav *et al.* 2024). We demonstrate the improvements in tracking by comparing KnotResolver to existing filament tracking tools.

## 2 Materials and methods

### 2.1 Reconstituting dynein-driven MT oscillations

**Figure 1.**
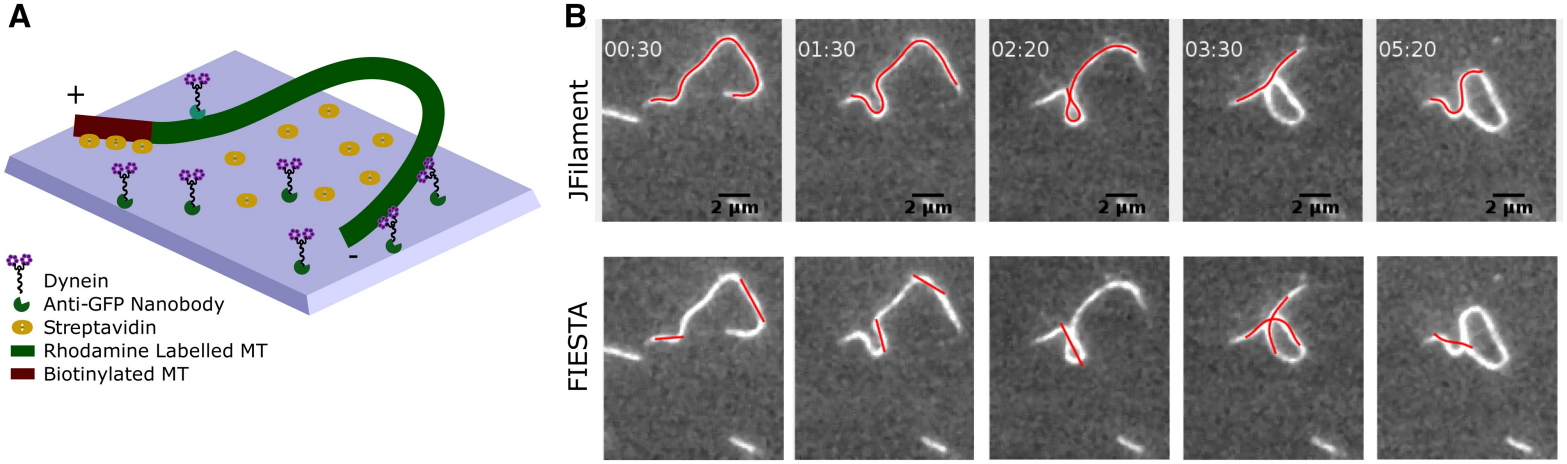
*In vitro* assay of filament oscillations and errors in detection using Gaussian fitting and active contours. (A) The schematic depicts the experimental setup of plus-end clamped MTs based on biotinylated tubulin bound to surface-immobilized streptavidin, combined with dynein motors immobilized by antibodies in a “gliding assay” that drives MT bending as described previously (Yadav *et al.* 2024). MTs are labeled with rhodamine-labeled tubulin for visualization in fluorescence microscopy. (B) Representative frames from a microscopy time series of the oscillations of rhodamine-labeled MTs (gray) oscillating over 5 min 20 s were segmented detected segments overlaid (red lines) based on either (top) an approach using open, active contours in JFilament (Smith *et al.* 2010) or (bottom) threshold and Gaussian fitting approach using FIESTA (Ruhnow *et al.* 2011). Scalebar: 2 μm, Time: mm: ss

The filament oscillation assays were performed in a flow chamber made by sandwiching double-backed tape between a slide and a coverslip. The chamber was coated with a 1:1 (molar ratio) mixture of anti-green fluorescent protein (anti-GFP) nanobody and streptavidin. The surface was blocked with casein to prevent non-specific attachment. Dynein was allowed to attach by incubating in the chamber followed by washes to remove unbound motors. The biotinylated MTs were added to the chamber and allowed to land followed by washes to remove the unbound MTs (Fig. 1A). Finally, a motility buffer containing anti-fade and 4 mM ATP was added to the chamber and MT movement was recorded. The setup was imaged using a 60× (NA 1.45) oil immersion lens using a motorized fluorescence microscope (Nikon TiE, Nikon Corp. Japan) with 10s time intervals for 10-20 min in a lexan enclosure with temperature maintained at 37°C (Okolab, Pozzuoli, Italy) as previously described (Jain *et al.* 2019, Yadav *et al.* 2024). Plus-end biotin-labeled MTs were prepared by first polymerizing filaments with a 1:4 molar ratio of rhodamine-labeled tubulin: unlabeled tubulin, centrifugation to remove monomers and plus labeling with a mixture of 1:4 rhodamine-labeled:biotinylated tubulin, with filaments then stabilized in taxol. The filament oscillation assays were performed in double-backed tape flow chambers made by sandwiching double-backed tape between a slide and a coverslip to form a chamber that was coated with the nanobody and streptavidin in a 1:1 molar ratio, followed by casein to block non-specific attachment. To this, the anti-GFP nanobody was added, then dynein-GFP, followed by multiple washes to remove unbound motors, biotinylated MTs, more washes, and finally motility buffer containing anti-fade and 4 mM ATP. The setup was imaged using a 60× (NA 1.45) oil immersion lens using a motorized fluorescence microscope (Nikon TiE, Nikon Corp. Japan) with 10s time intervals for 10–20 min at 3°C in a temperature-controlled Lexan enclosure (Okolab, Pozzuoli, Italy) as previously described (Yadav *et al.* 2024).

### 2.2 Simulating oscillatory filament dynamics and the effect of image “noise”

#### 2.2.1 Simulations

We performed simulations of oscillatory wave-like dynamics of simulated MTs based on a stochastic agent-based simulation engine for motor–cytoskeleton interactions, Cytosim (Nedelec and Foethke, 2007), based on a previously described 2D dynamics model of individual plus-end clamped MTs in a gliding assay (Yadav *et al.* 2024). We systematically varied MT lengths from 5 to 50 μm and motor density over 12, 25, 50, and 100 motors/$\mu m^{2}$. Each simulation was performed for 5 min and 120 frames were saved. The objective was to generate skeleton contours of varying lengths, comparable to experiments.

#### 2.2.2 Adding “noise” to simulated images

The simulated skeletons were dilated by a diamond-shaped structuring element of size 2 and the dilated skeletons were convolved with a Gaussian point spread function (size = 3, sigma = 2). We introduced noise of varying signal-to-noise ratio (${\mathrm{SNR}}_{db}$) to the simulated data. The noise model is Gaussian with a specified noise power level $P_{\mathrm{noise}}$. The desired $P_{\mathrm{noise}}$ is related to SNR by the relation:

(1) $$P_{\mathrm{noise}}=P_{\mathrm{signal}}/\mathrm{SNR}$$

where $P_{\mathrm{signal}}$ is equal to the variance of intensity of the original image. *SNR* in linear scale is related to $SNR_{db}$ by the following relation

(2) $$\mathrm{SNR}=10^{\frac{SNR_{db}}{10}}$$

The $SNR_{db}$ values used were 5, 10, 20, and 30. The generated Gaussian noise is computed as *Noise*:

(3) $$\mathrm{Noise}∼\sigma_{\mathrm{noise}}*N(0,1)$$

where, N is a normal distribution with zero mean and the $\sigma_{\mathrm{noise}}$ is equal to

(4) $$\sigma_{\mathrm{noise}}=\sqrt{P_{\mathrm{noise}}}$$

The *Noise* is added to the original image to get a noisy image with specific $P_{\mathrm{noise}}$.

### 2.3 Quantifying filament detection accuracy

The simulated dataset described previously was used to create benchmarks to assess the proposed filament tracking method. We quantified detection accuracy using the simulated dataset as a “gold standard” and some typical measures used in image-analysis accuracy using two measures: (i) Sørensen–Dice Index for segmentation and (ii) Fréchet distance to compare two curves.

#### 2.3.1 Dice index

The Dice score or Sørensen–Dice index (Dice 1945; Sorensen 1948) takes into consideration TP: true positives, FP: false positives, FN: false negatives, and TN: true negatives and combines them as follows:

(5) $$\text{Dice index}=\frac{2\times \text{TP}}{2\times \text{TP}+\text{FP}+\text{FN}}$$

True positives are defined as segmented pixel indices that are within a threshold distance (2 pixels) from the ground truth annotations. The choice of the threshold was based on the point spread function used to convolve the filament skeletons to resemble experimental images. False positives are segmented pixels that are not within this threshold. Ground truth pixels that were not detected as TP are counted as false negatives.

#### 2.3.2 Fréchet distance

This metric computes the minimum distance between two curves that is sufficient to traverse their separate paths (Fréchet 1906, 1924) using a MATLAB implementation of the discrete Fréchet distance. This provides the maximum separation in distance metric between the two curves. The metric is useful for comparing the overlap of two curves, while taking into account the proximity of indices between the two curves. For a pair of duplicate curves, Fréchet distance should be zero, which indicates a perfect overlap and identical ordering of pixel indices between two curves. Fréchet distance is computed between the indices of input curve $I(t)=(I_{x}(t),I_{y}(t))$ and the resolved curve $R(t)=(R_{x}(t),R_{y}(t))$ obtained from the KnotResolver pipeline. Here *t* signifies the orientation or ordering of the two pixel indices in their respective curves. The Fréchet distance is computed as follows:

(6) $$F(I,R)=\underset{\alpha ,\beta }{\inf}\underset{t}{\max}\{\rho (t,\alpha ,\beta )\}$$

where ρ(t,α,β) is the distance function defined as:

(7) $$\rho (t,\alpha ,\beta )=\sqrt{{\left(I_{x}(\alpha (t))-R_{x}(\beta (t))\right)}^{2}+{\left(I_{y}(\alpha (t))-R_{y}(\beta (t))\right)}^{2}}$$

and α and β are re-parameterizations of the curves I(t) and R(t), respectively.

### 2.4 Analysis of filament contours

All analysis was performed in MATLAB R2020b (Mathworks Inc., Natick, MA, USA). We calculate the tangent, normal, and curvature vectors for each smoothed contour by using the Frenet–Serret formulas (Mate 2017). Smoothing of the *x* and *y* coordinates is performed using the Savitzky–Golay filter (Savitzky and Golay 1964). The end-to-end distance of the curve is computed as Euclidean distance between the tip and end of each filament. All results are visualized by plotting the curve with color interpolation, which indicates the arrangement of contour indices from tip to end.

### 2.5 Code availability

The tool was written in MATLAB2020b (Mathworks, Natick, MA, USA) and uses functions called from the “Signal Processing Toolbox,” “Image Processing Toolbox,” “Statistics and Machine Learning Toolbox,” and an optional “Parallel Computing Toolbox.” The code for the filament bending analysis has been made OpenSource and is available at the Github URL https://github.com/CyCelsLab/MTKnotResolver, together with an example time series with optimized segmentation parameters.

## 3 Results

### 3.1 Filament looping and cross-over detection: limits of existing tools

We have recently demonstrated MTs can undergo waves of high curvature and oscillate in a modified *vitro* “gliding assay,” where plus-ends of MTs were clamped to the glass substrate, while a surface-immobilized minus-end directed motor, dynein, generated the transport force (Yadav *et al.* 2024) (Fig. 1A). The filaments undergo wave-like millihertz (slow) oscillations in a motor-density and filament length-dependent manner. In order to quantify filament contours from such image time-series data, we applied multiple standard image analysis tools for segmenting filaments in “gliding assays” of which we describe the result of using the two more widely used: JFilament and FIESTA. JFilament (Li *et al.* 2009, Smith *et al.* 2010), based on a robust active contour approach, resulted in filament detections only when they were not looped, but once filaments underwent self-intersections, the method took what appeared to be the shortest path [Fig. 1B (top)]. This relates to the nature of the active contour method used. FIESTA (Ruhnow *et al.* 2011), on the other hand, is based on Gaussian profile fitting to improve threshold-based segmentation with filament tracking broadly based on distance minimization. The outcome of using the same dataset resulted in detection only of the straight segments of filaments, with highly curved regions of the same filament fragmented into multiple straight segments [Fig. 1B (bottom)]. While tuning multiple parameters of the code did change the results slightly, it did not lead to a qualitative improvement, due perhaps to the inherent assumption of straight rod geometry built into the method. Thus, we proceeded to address both these limitations of the existing approaches—straightness assumption and path length optimization—to develop a novel approach.

### 3.2 Resolving self-intersecting “knots” in filament contours by minimizing paths using directed graphs

**Figure 2.**
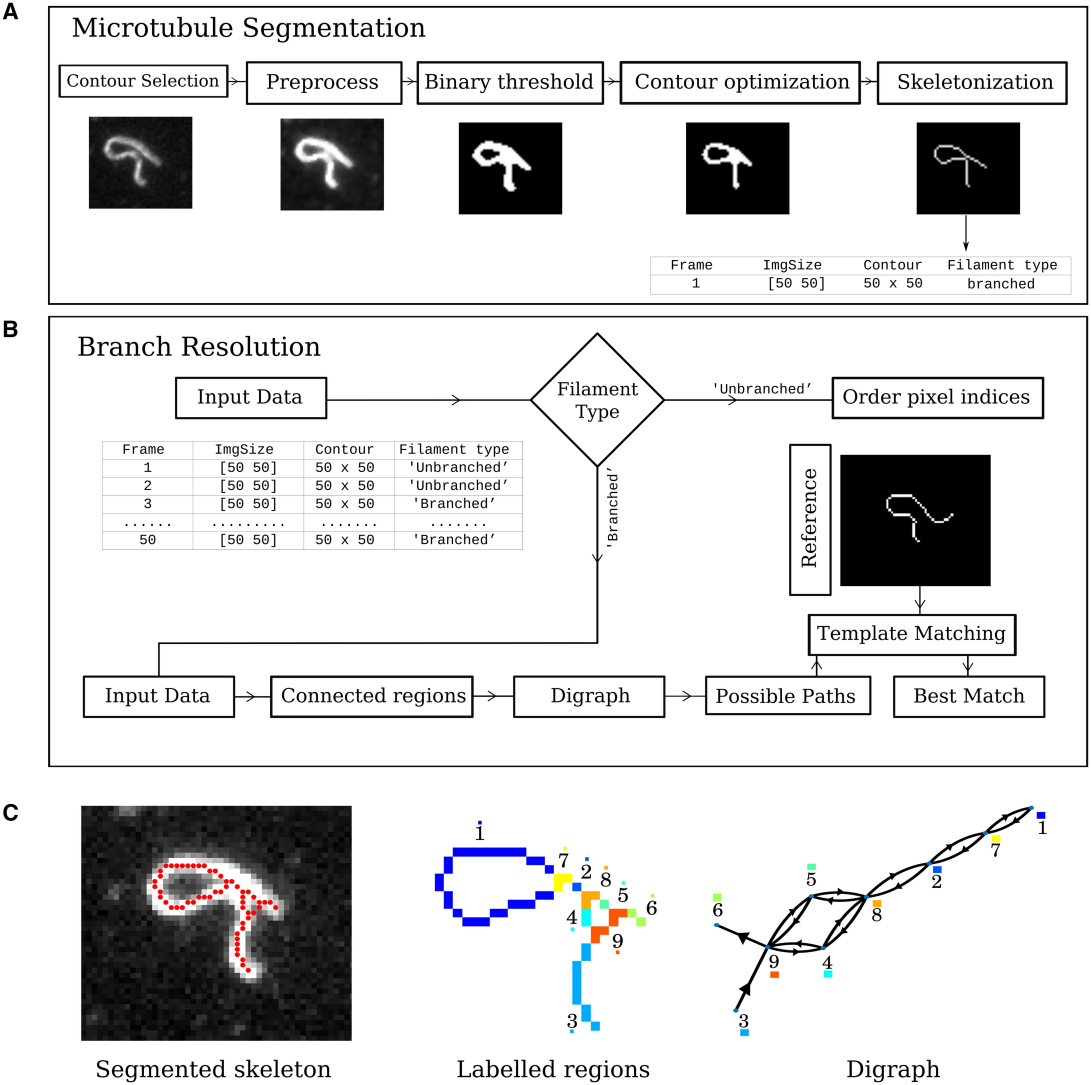
Algorithmic flow diagram of graph-based resolution of self-intersecting filament image time series. (A) “Segmentation and branch finding”: In a frame-wise manner, images are binarized by thresholds. These binary objects are used to seed active contours to smooth the curve. The contour is skeletonized to 1 pixel width using medial axis transform, and pixels with 4 or more neighbors are labeled as branched, suggesting a self-intersection. (B) “Branch resolution”: The skeleton with sequential pixels is used as an input labeled as either “branched” or “unbranched.” Branched contours are mapped to a directed graph (digraph) with branch points as vertices connected to each other by segments of the contour as edges. Paths are calculated using a two-step coarse and refined approach to reduce complexity. Graph-paths are mapped back to possible contours, whose distance is then minimized by comparison to the last unbranched contour (template), to find the best match. (C) (left to right) A representative grayscale image (gray) segmented resulting in a skeleton contour (single pixel wide contour), that is classified into regions (numbered) that connect branch points. These regions are mapped to edges and branches to vertices, with both directions possible based on a user-input start point, to produce a directed graph

The “KnotResolver” pipeline consists of the following steps: (i) “Segmentation” used to extract skeletons of a single MTs (Fig. 2A). Segmentation parameters are interactively set by visually inspecting the output with the optimal values saved in a CSV file. (ii) “Branch identification” is called after the segmentation optimization script that identifies filament self-intersections (Fig. 2B). (iii) “Mapping ‘knots’ to graph”: converts the elements into a graph representation, (iv) “Template matching” to reconstruct the correct filament geometry by path minimization to previous time frames (Fig. 2B and C).

**Figure 3.**
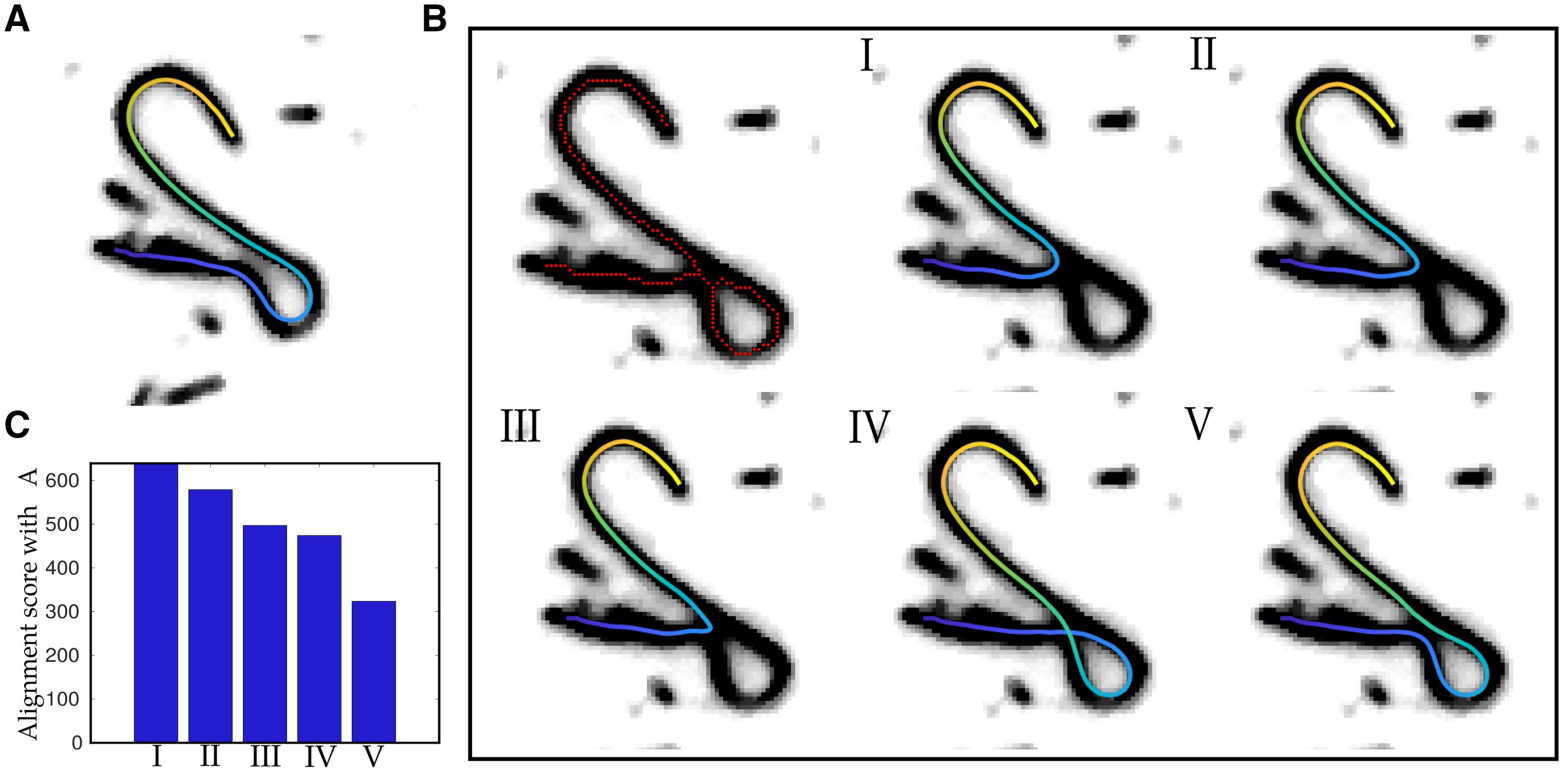
Resolve the knot by minimizing distances between template and paths generated by the graph. (A) A representative image of a bend microtubule at time *t*, when it does not have any knots (unbranched) with the segmented contour. The intensity of the contour maps the position index along the contour. (B) The microtubule in frame *t+*δ*t* showing a knot (branched) as seen from the contour (red line). (I–V) The possible paths from the start to the other end are overlaid as contours with the position-index in color. Blue: start, yellow: end. δt: interval between frames. (C) The alignment score is calculated for all paths (I–V) compared with the unbranched path from the previous time frame based on dynamic time warping. The path with the lowest score is then the optimal

- “Segmentation”: images were first pre-processed to enhance contrast by saturating the top and bottom 1% of intensity values and median filtering using a 3 × 3 kernel to smooth the intensity fluctuations. The pre-processed image stack was converted to binary (black–white) by Otsu’s method of finding the threshold (Otsu 1979) and the segmented image with filament contours used as the initial seed for an energy-based active contour model based on Chan and Vese’s method (Chan and Vese, 2001). This improves the segmentation by optimizing for the filament boundary. Finally, the optimized binary contour is skeletonized using the medial axis transform (Lee *et al.* 1994) to obtain one-pixel width filament contours, which are stored in a structured array for all frames in the image stack [Fig. 2A and C (left)].
- “Branch identification”: Skeletons obtained in the previous step are tested for branch points in a neighborhood of 8 (Moore neighborhood) by testing whether the summation of individual pixel values ($I_{nb}$ = either 0 or 1) satisfies $\Sigma_{nb}^{N}(I_{nb})\geq 4$. The central pixel (nb=5) has a value 1, by virtue of being on the skeleton. In cases where there are no branch points in a segmented skeleton, the correct orientation of the filament indices is determined using the geodesic distance algorithm (Soille *et al.* 1999) implemented using the *bwdistgeodesic* function in MATLAB (Mathworks Inc., Natick, MA, USA). Filament end points are provided as a seed to the function. When the skeleton is identified as being a “knot,” i.e. with branches, we proceed to use a graph representation, as described in the following section.
- “Mapping *‘*knots*’* to graphs”: a skeleton containing branch points is represented as a directed graph G=(V,E) consisting of vertices *V* and edges *E*, with the direction given by a chosen start point. They are labeled as either branch ($V_{b}$) or filament segment ($V_{f}$) (Fig. 2B). A branch vertex is identified by connected pixels that satisfy the branch-point criterion, while the remaining regions are labeled as filament vertices. Edges *E* are the connectivity between unique label groups, forming connecting pairs of $V_{b}$ and $V_{f}$ vertices (Fig. 2C). All edges are bidirectional, i.e they can be $E(V_{b},V_{f})$ or $E(V_{f},V_{b})$, enabling traversal in either direction, except for start and end vertices where traversal is limited to a single direction. Instances where a filament merges with itself are represented as loops, in the graph, i.e. where heads and tails coincide (Bang-Jensen and Gutin 2001), e.g. for the pair of edges: E(1,7) (Fig. 2C).
- “Template matching”: a branched skeleton thus represented as a graph $G=(V_{i},E_{i,j})$, where $V_{i}$ denotes the set of vertices and $E_{i,j}$ denotes the set of bidirectional edges, where *i* and *j* are indices of branched or filament vertices. The graph structure is determined by the number of unique branch points, which either indicate intersections with other filaments or an instance where the filament forms a loop. We generate all possible path combinations between the start and endpoint by traversing every edge connecting vertices $V_{f}$ (filament vertex) and $V_{b}$ (branch vertex). For graphs that lack an end point, the path ends at the last visited node, after all nodes are visited once. Since the number of paths can increase exponentially with the number of nodes, we optimized the number of possibilities generated at each step to capture filament paths correctly, based on comparison to the “ground truth,” obtained from manual analysis. Knot resolution is based on minimizing the difference to a resolved skeleton, which is the template (Fig. 3A). We have implemented a multi-step approach: (i) coarse identification of key vertices using Euclidean distance matching, (ii) path generation to output possible paths between the start and end point, and (iii) template matching of possible paths to the previous resolved contour. (i) Identifying key vertices: Euclidean distance scores are of vertices compared to the template that help identify connections with minimal distance to a reference—to identify the three vertices with minimal distance to the template ($V_{m=1to3}$). These vertices are used to limit the search and significantly reduce the number of paths generated. (ii) Path generation: multiple paths are generated between a start node and a defined end vertex (Fig. 3B). The first combination of path is generated by taking into account the template found in the previous step. The remaining paths are generated by finding number of combinations between the fixed start point and all of the remaining vertices sequentially. The number of paths generated at every combination is limited to 10 to avoid exponential growth of possibilities. (iii) Template matching: all possible paths are converted to contours, and an alignment score is assigned using dynamic score warping, DTW (Sakoe and Chiba 1978). The score is calculated as the cumulative cost function D(i,j) between a selected contour $S(x_{i},y_{i})$ and $R(x_{j},y_{j})$ given by:
  (8) $$\begin{array}{c} D(i,j)=\left|S(x_{i},y_{i})-R(x_{j},y_{j})\right|\\ +\min\left(D(i-1,j-1),D(i-1,j),D(i,j-1)\right) \end{array}$$

where *i* is the reference index and *j* the template index. This score is compared among the possible paths, and the path with the minimal score is selected (Fig. 3C). Once a match is found, pixel indices are iteratively ordered, along the best found path using the geodesic distance (Paliwal *et al.* 1982).

The pipeline of the implementation is optimized for single filaments, but the input file can be used to batch process multiple ROIs. This proceeds with the user creating a *myParams.csv* file with details of the file names and paths on each line—a new line indicates a new file. This is run as an input to *KnotSegmenter.m*, which after graphic user interface (GUI) based interactive optimization of segmentation parameters, results in an output *myParams_optimized.csv* file. This has the optimized segmentation parameters for each ROI, and is used as an input by *KnotResolver.m* to batch process the ROIs.

### 3.3 KnotResolver has Sub-pixel filament contour detection accuracy and is robust to image “noise”

**Figure 4.**
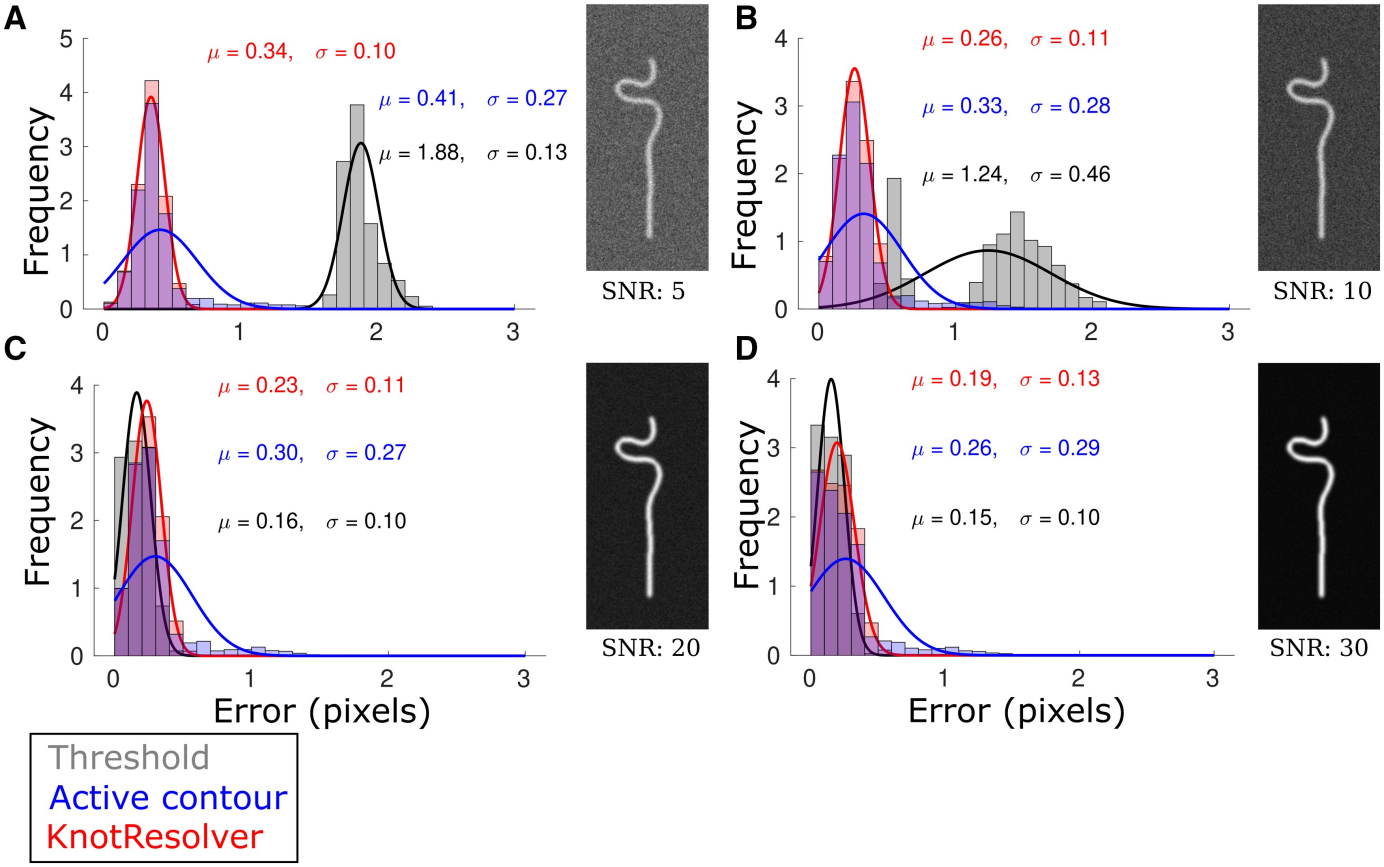
Position detection error compared between segmentation methods with increasing noise. (A–D) The segmentation error in pixels for each filament compared to ground truth from simulations is plotted as a frequency histogram for the following methods: (i) threshold, (ii) active contour, and (iii) KnotResolver (legend). The inputs were simulated filament images with increasing signal-to-noise ratios (SNR) (A) 5, (B) 10, (C) 20, and (D) 30. The mean (μ) and SD (σ) of a Gaussian fit to each error-histogram is noted (*n = 2880*). The images are representative filaments with increasing SNR, with salt and pepper noise resulting in low SNR

In order to assess the segmentation accuracy of our method, we created images of simulated time series of filaments that undergo cross-overs based on plus-ends clamped and minus-end directed motors in a modified “gliding assay,” as described previously (Yadav *et al.* 2024) and briefly summarized in Section 2. These simulated MT coordinates in time series were plotted as binary skeletons to provide the “ground truth” and processed to resemble experimental images of filaments by dilation with a diamond-shaped structuring element of Size 2 and convolution with a Gaussian point spread function (Size: 3, σ: 2) centred around the central pixel. Salt-and-pepper noise of different intensities was added and the SNRs were calculated. The error in position detection is calculated as the minimal distance between the segmented skeleton and the ground truth pixel indices. “KnotResolver” was then used to segment the images of bent filaments, and compared to a simple intensity threshold and active contour-based approach, when the SNR increased from 5 (Fig. 4A), 10 (Fig. 4B), 20 (Fig. 4C), to 30 (Fig. 4D). We find the mean pixel error in position detection of “KnotResolver” and active contour methods remains sub-pixel for the range of SNR as seen by the Gaussian peak from fitting (μ:mean, σ: SD), but simple threshold-based methods show errors exceeding few pixels for SNR 5 and 10. In image-frames where the filament contour self-intersect, only KnotResolver can correctly identify contours, with both segmentation and active contour methods failing (Fig. S1A). The outputs of “KnotResolver” are robust for SNR ranging from 30 to 5, in contrast to threshold-based methods and active contours that are sensitive to the same SNR range, as quantified by the Dice Score, as described in detail in Section 2 (Fig. S1B). Threshold-based methods as highlighted are sensitive to low SNR, while active contour methods are sensitive to rapidly changing geometry that throws off the initial seed and leads to filament shrinkage. The combination of these two methods, implemented in KnotResolver, is robust to both low SNR and rapidly changing filament positions, which is key for tracking single filaments showing sustained oscillations.

In order to identify possible limitations of our method and solve them, we tested the effect of multiple self-intersections that arise in simulations with increasing filament length and motor density and find Dice score decreasing after ∼60 frames in MTs with lengths >20 μm (Fig. S2A). As a measure of the geometric shape, we also use the Fréchet distance score between segmentation result and ground truth and find a comparable increase in distance indicating drop in accuracy of segmentation for long MTs at high densities (Fig. S2B). To address this limitation, we have implemented a manual restart, which can be used when the code encounters problematic frames using user-input to interactively map the path in the graph structure and correct the errors (Fig. S2C). In those time series where the first frame itself is self-intersecting, the user can manually initialize the correct path of the contour elements (labeled regions) by first running *KnotSegmenter.m* to obtain the skeleton, and then running *KnotResolver.m* with the entry “ManualCheck” set as [p], where *p* = 1 to *N*, the frame number where manual intervention is required (Fig. S3A). Running the code pops up an interactive plot with the labeled regions (Fig. S3B), which the user can click on segments sequentially to provide the path of resolution (Fig. S3C), which in turn resolves the knot (Fig. S3D).

In summary while sub-pixel accuracy can be achieved by other methods alternative to the proposed KnotResolver pipeline, the higher Dice score in cases of self-intersections demonstrates both high accuracy and robustness to noise. The addition of a manual override also offers the user control over the path selection in exceptional cases.

### 3.4 KnotResolver outperforms existing methods for MT filament tracking

**Figure 5.**
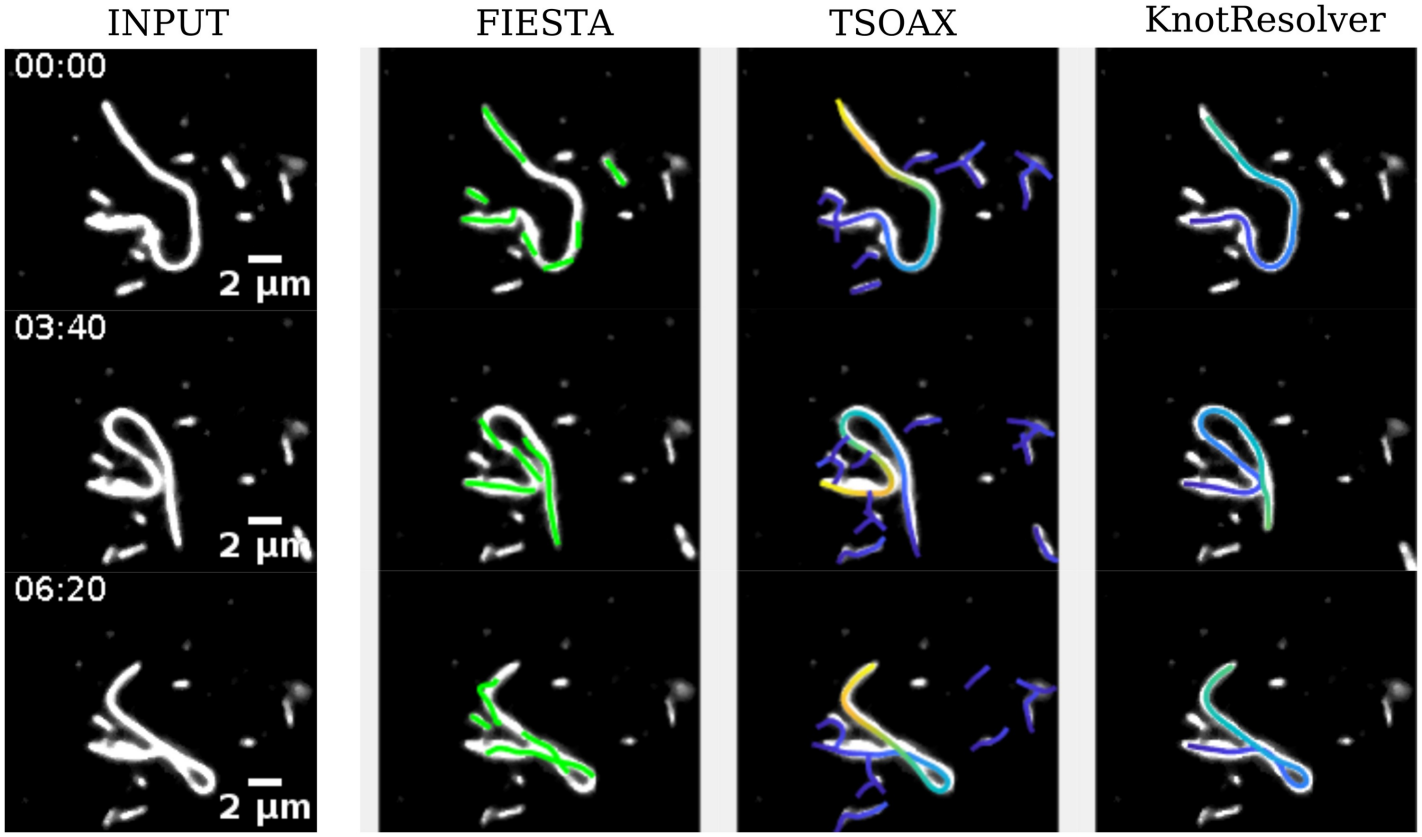
Comparing KnotResolver to FIESTA and TSOAX. (INPUT) A representative time series of labeled MTs undergoing bending and buckling forming a knot was used as input and tracked using FIESTA (Ruhnow *et al.* 2011), TSOAX (Xu *et al.* 2019), and KnotResolver. Parameters used for (i) FIESTA were FWHM = 1000 nm, proportion curved filaments = 25%, minimum track length = 1 frame, maximum break = 4, (ii) for TSOAX default parameters were used with manual input of the minimum and foreground intensity value and (iii) KnotResovler were intensity threshold = 0.46, number of iterations for active contours = 10, contraction bias = 0.4, and smoothing factor for active contours = 0.2, with no manual corrections. Scale bar: 2 μm. Time: mm: ss

We compare of our approach to resolving self-intersecting filaments in terms of segmentation and tracking to two widely used computational tools—FIESTA (Ruhnow *et al.* 2011) and TSOAX (Xu *et al.* 2019). FIESTA is based on intensity thresholding and Gaussian fitting of contours to achieve sub-pixel localization accuracy. Filament intersections are semi-automatically handled with cross-point identification followed by linking based on a minimum angle criterion. TSOAX, on the other hand, combines stretching open active contours based software (SOAX) (Xu *et al.* 2015) with tracking. This is achieved through automatic initialization of multiple open curves during the detection stage, which elongate and stop at filament intersections and tips. Intersections of curves are addressed by identifying T-junctions linked through a temporal local matching step. In order to compare the outcomes, we use an experimental microscopy time series of MT filaments in a clamped dynein-driven gliding assay. The filaments are observed to change from straight, to highly curved, to self-intersecting filaments (Fig. 5). While the output from FIESTA demonstrates only straight segments are detected, TSOAX can identify curved filaments, but cross-overs are incorrectly detected. The output of KnotResolver is the only one that allows us to correctly identify curved sections and maintain the identity of the segments at cross-over.

**Figure 6.**
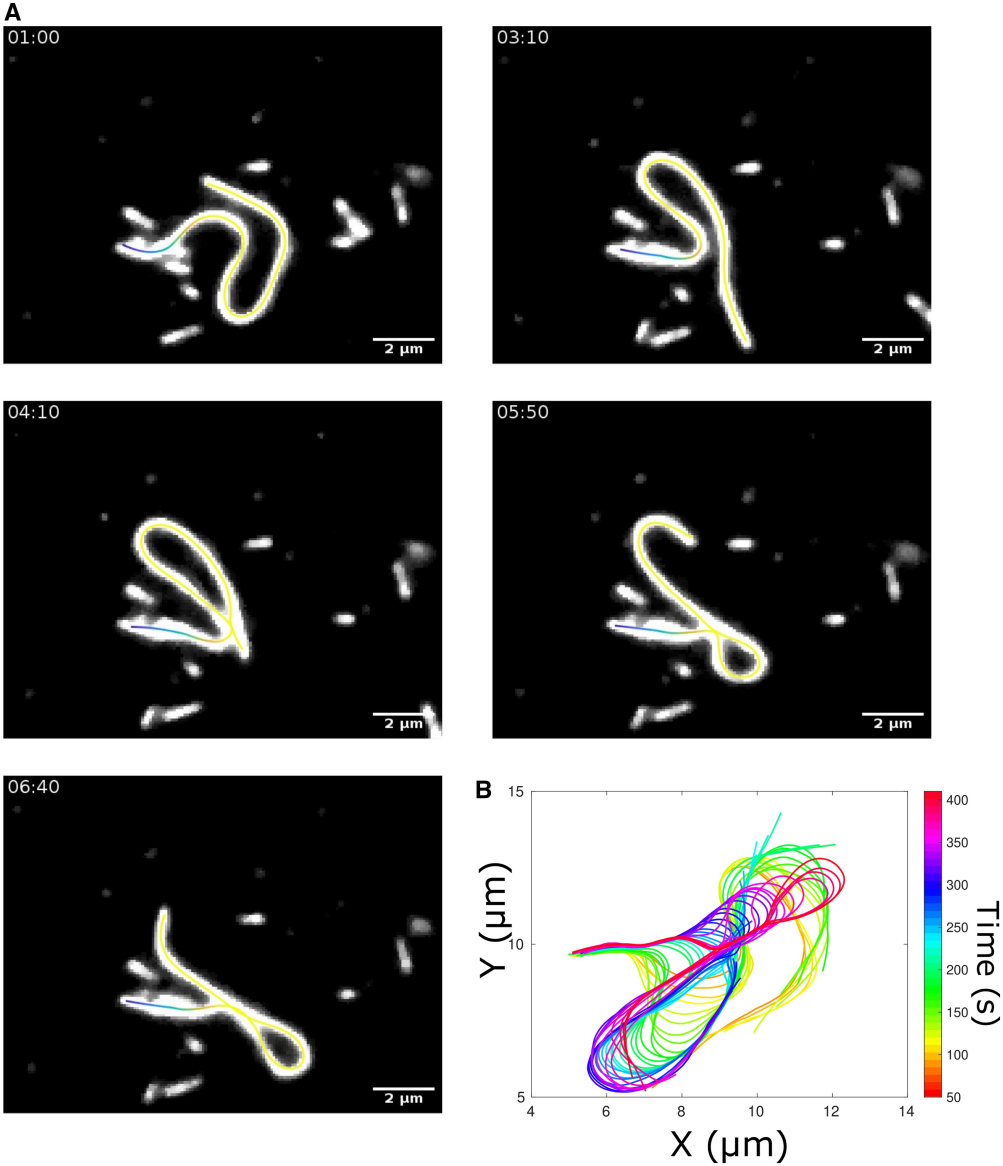
Representative time series of filament with tracked contours. (A) The fluorescence time series of bent and crossing over filaments are overlaid contour segmented using proposed pipeline. The skeleton is color-coded along its length (form tip to end). Scale bar: 2 μm. Time: mm: ss. (B) The filament contours from successive frames are projected showing the traveling wave of curvature. Colorbar: time

**Figure 7.**
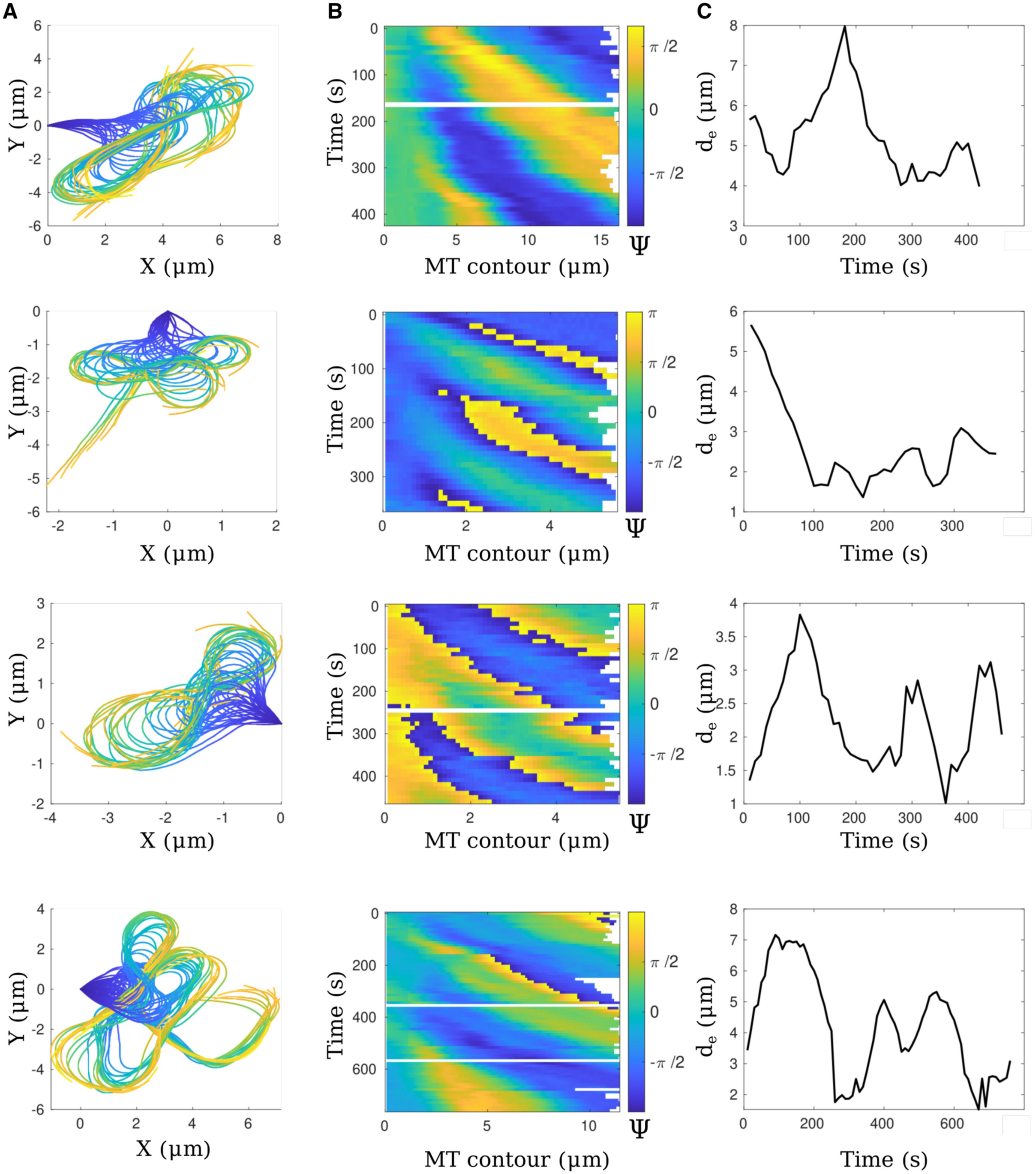
Contour analysis in time of representative experimental filaments. Four filaments showing beating mobility patterns are shown with (A) the montage of the skeletal line, coded along its length (fixed-tip to end), (B) a 2D plot of tangent angle along the microtubule length (μ*m*) with colorbar representing the angle in radians and (C) the end to end distance (*y*-axis) plotted with time in seconds (*x*-axis)

**Figure 8.**
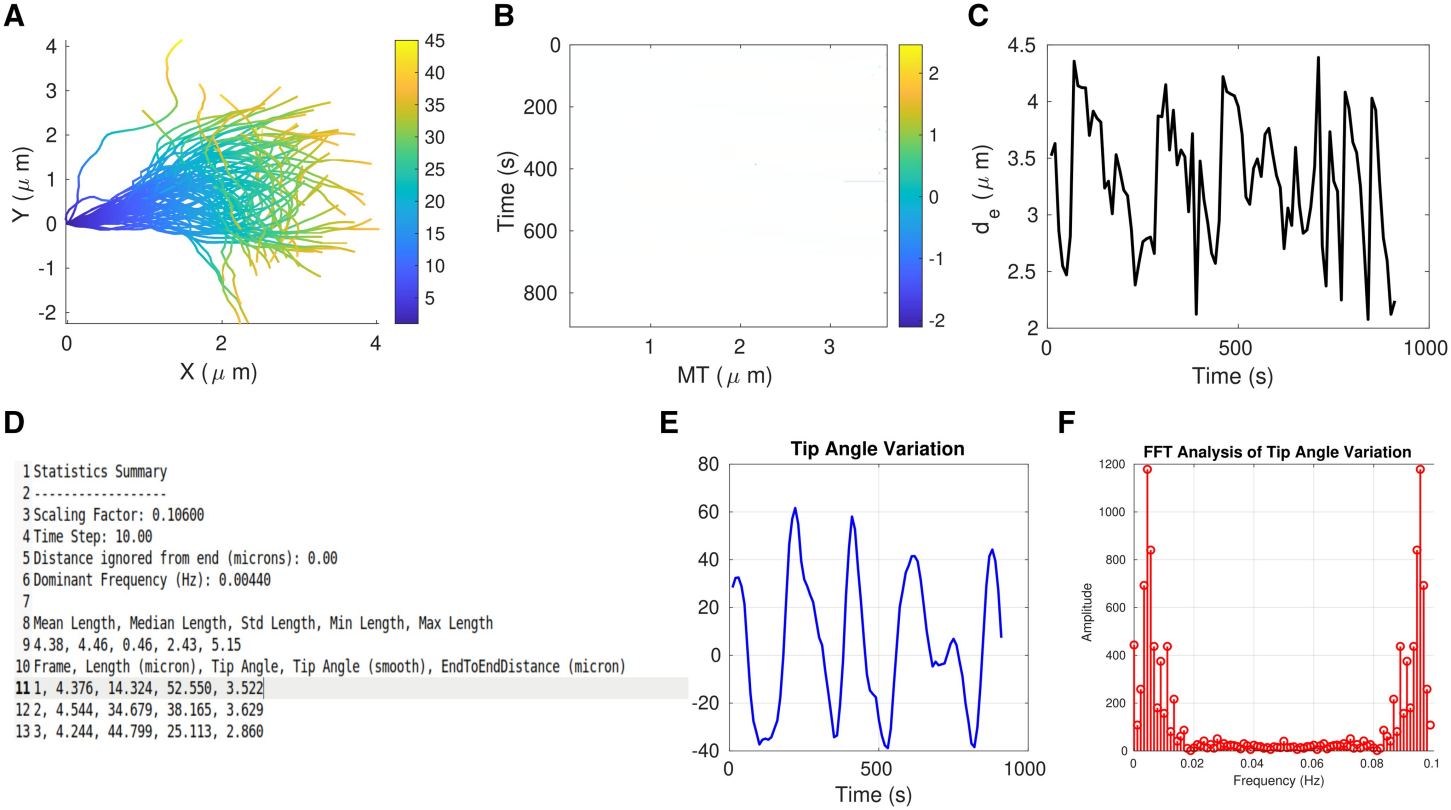
KnotResolver outputs. (A) The time-projected contours detected by KnotResolver of a tracked filament is plotted. Colorbar: Map of position from the immobile (blue) to the free end (yellow). (B) The kymograph of the tangent angle (color) with time (*y*-axis) and position along the contour (x-axis). Colorbar: angle in radians. (C) End-to-end distance between the fixed tip and the free end of the pinned filament (*y*-axis) plotted as a function of time (*x*-axis). (D) The text file output (*statsSummary.txt*) provides a summary of input parameters followed by frame-wise information about (left to right) frame number, filament length, tip angle, and the end-to-end distance. (E) The tip angle plotted with time is used to calculate (F) the power spectrum by FFT analysis (using the MATLAB command *fft*) of tip-angle dynamics. The graph represents the amplitude (*y*-axis) as a function of frequency (*x*-axis)

We therefore proceeded to apply the KnotResolver pipeline to a representative experimental time series of clamped filaments undergoing periodic wave-like oscillations and demonstrate that it outputs a smooth contour (Fig. 6A, Video 1). The time-resolved data of filament dynamics projected demonstrates the wave-like oscillations (Fig. 6B). We test the tool on multiple such time series, resulting in correct segmentation of experimental inputs, as seen in time-projected contours (Fig. 7A). Since the wave-like propagation is flagella-like, we use a similar measure to quantify the dynamics in terms of the local tangent angle (Ψ) dynamics along the filament length in a kymograph and find the curvature to propagate base-to-tip in a periodic manner (Fig. 7B). We also plot the distance between the filament ends ($d_{e}$) in order to examine whether the angular dynamics are reflected in tip position movement. The periodic change in the end-to-end distance mirrors the periodic angular movement of the filament (Fig. 7C). The oscillatory period from peak-to-peak in both angular plots and the end-to-end distance is a few millihertz, similar to what we had previously reported for clamped MTs in dynein gliding assays (Yadav *et al.* 2024). This low frequency oscillation is also consistent with theoretical expectations based on filament bending rigidity and motor mechanics. The tool results in multiple plots (Table S2) and a text file with frame-wise statistics of tracked filaments (Fig. 8). This can be used to further analyse biologically meaningful properties like filament rigidity and time-dependence of the dynamics and their relation to motor-filament interactions.

Thus, we demonstrate the utility of the directed graph based pipeline KnotResolver in automated quantification of filament time series that could be used for other systems.

## 4 Discussion

Here, we have demonstrated the limitations of existing methods and softwares to detect and track self-intersecting filaments in microscopy time series. We describe a computational image-processing pipeline, KnotResolver, based on intersection detection, mapping to directed graphs, contour tracing in two steps and distance minimization to a template. We demonstrate that this approach results in sub-pixel position detection and high Dice scores in identification, robust to image noise. In comparison to existing tools, we demonstrates KnotResolver overcomes their limitations and when applied to experimental fluorescence microscopy time-series images of a “gliding assay” where the filament end is clamped, can allow us to automatically quantify geometry and frequency of oscillations.

Similar to many such software developed, KnotResolver has some parameters that need to be user input (Table S1). These are optimized for MTs in a dynein gliding assay with pinned filaments. A further test of the utility of this code could be to analyse time series in more complex backgrounds such as inside cells where compressive forces result in buckling instabilities seen in MT filaments (Brangwynne *et al.* 2006). We also expect that actin-based gliding assays with typically bent filament with complex curvature, due to the lower persistence length compared to MTs (Gittes *et al.* 1993), could also be tracked using this tool. Indeed surface-defects in myosin-driven actin gliding assays have been previously reported to also result in qualitatively comparable structures (Bourdieu *et al.* 1995). We expect the use of KnotResolver to extend to multiple cytoskeletal filament analysis.

In conclusion, our OpenSource computational image analysis tool that uses a novel directed graph-based mapping to resolve self-intersections of cytoskeletal filaments from both simulated and experimental data could be useful to automate the segmentation and tracking of “gliding assays” with complex geometries, but also flagellar dynamics and possibly filament oscillations in cells.

## Supplementary Material

btae538_Supplementary_Data

## Data Availability

The code for the filament bending analysis has been made OpenSource and is available at the Github URL https://github.com/CyCelsLab/MTKnotResolver
