# Supplementary material for: KnotResolver: tracking self-intersecting filaments in microscopy using directed graphs
Source: Bioinformatics. 2024 Sep 3;40(9):btae538. doi: 10.1093/bioinformatics/btae538 (PMC11483626; doi:10.1093/bioinformatics/btae538)
Supplement: btae538_Supplementary_Data [file btae538_Supplementary_Data.zip › SUBMIT-SupMat-Athale-bioinfoFormat.pdf]

Supplemental material

1. Supplemental tables
2. Supplemental figures
3. Supplemental movies

Supplemental tables

Parameter	Typical value	Description
segThresh	0.46	Threshold for intensity segmentation
segIteration	10	Number of iterations for active contour optimization
segContract	0.4	Contraction bias for active contours
segSmooth	0.2	Smoothing factor for active contours

Table S1. Parameters of KnotResolver. The parameters typically used for microtubule bending and looping image-time series that can be modified by the user for other input image types.

Parameter	Figure output	Description
Oscillation Frequency (Hz)	TipAngle	Oscillation frequency calculated from the tip angle variation in the time series.
Contour Length (μm)	CountourOverlay	Contour or filament calculated as the sum of distances between pixels and re-scaled with the input scaling factor.
Tip Angle (degree)	TipAngle	Tip angle is calculated between the fixed tip and the endpoint
End to End Distance (μm)	End2End	Straight line distance between the first and the last pixel
Tangent Angle (degree)	TangentAngleKymograph	Tangent angle calculated along the contour for every frame

Table S2. Quantification outputs from KnotResolver. The variables with frame-wise dynamics that are plotted as outputs from KnotResolver as .pdf files.

Supplemental figures

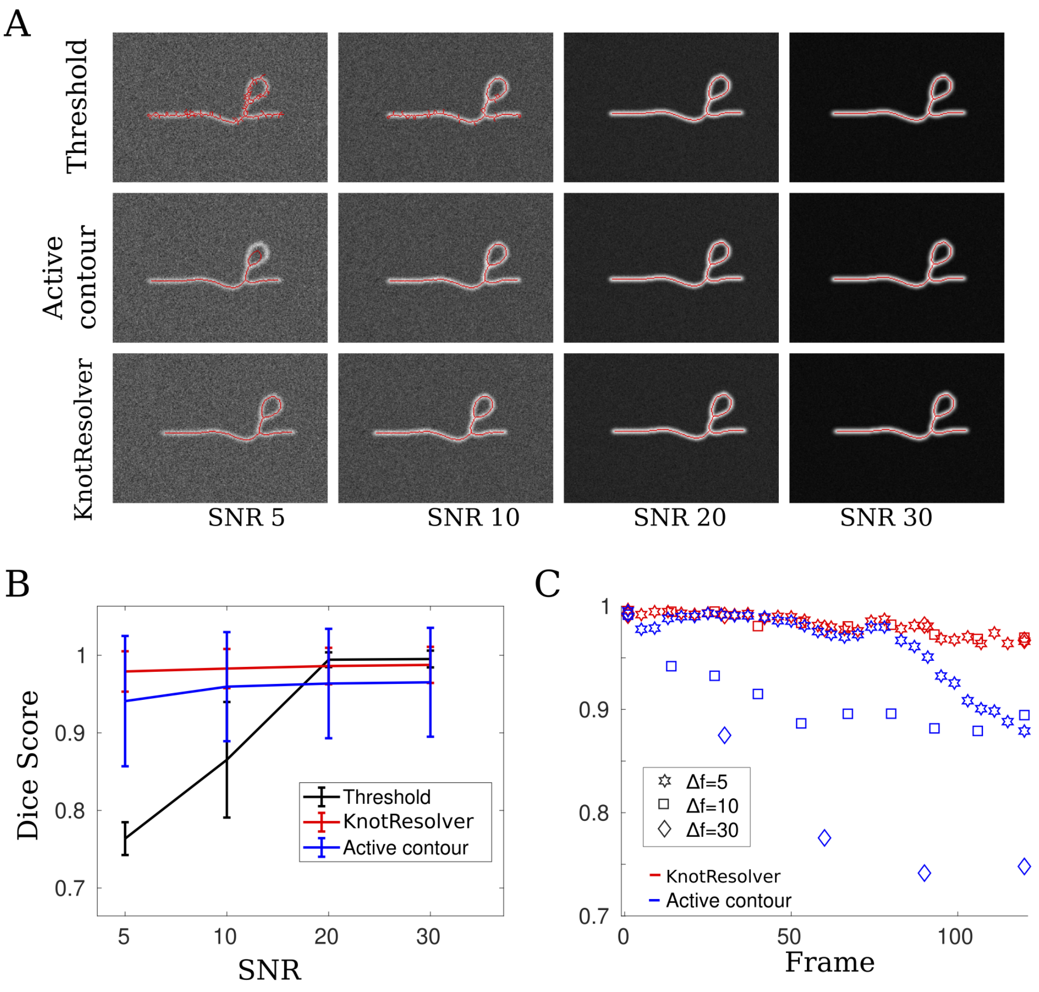

Figure S1. Dice score of segmentation accuracy by KnotResolver with increasing 'noise' compared to simpler approaches. (A) Montage of segmentation outputs (shown in yellow) overlaid on the input image for the three compared approaches at different SNR. **(B)** Filament segmentation accuracy is compared using the Dice Score (Equation 6). Three alternative approaches were tested: threshold-based (black), active contour (blue) and KnotResolver (red). Error bars indicate the standard deviation around the mean. **(C)** Impact of increasing time step between frames on segmentation accuracy. Segmentation accuracy is compared between KnotResolver (red) and the active contour (blue) method for three frame interval values Δ f = 5 (✡), 10 (□) and 30 (◇). Mean Dice scores are computed for every frame across all time series. The analysis involves a total of n = 24 time series, each consisting of 120 frames.

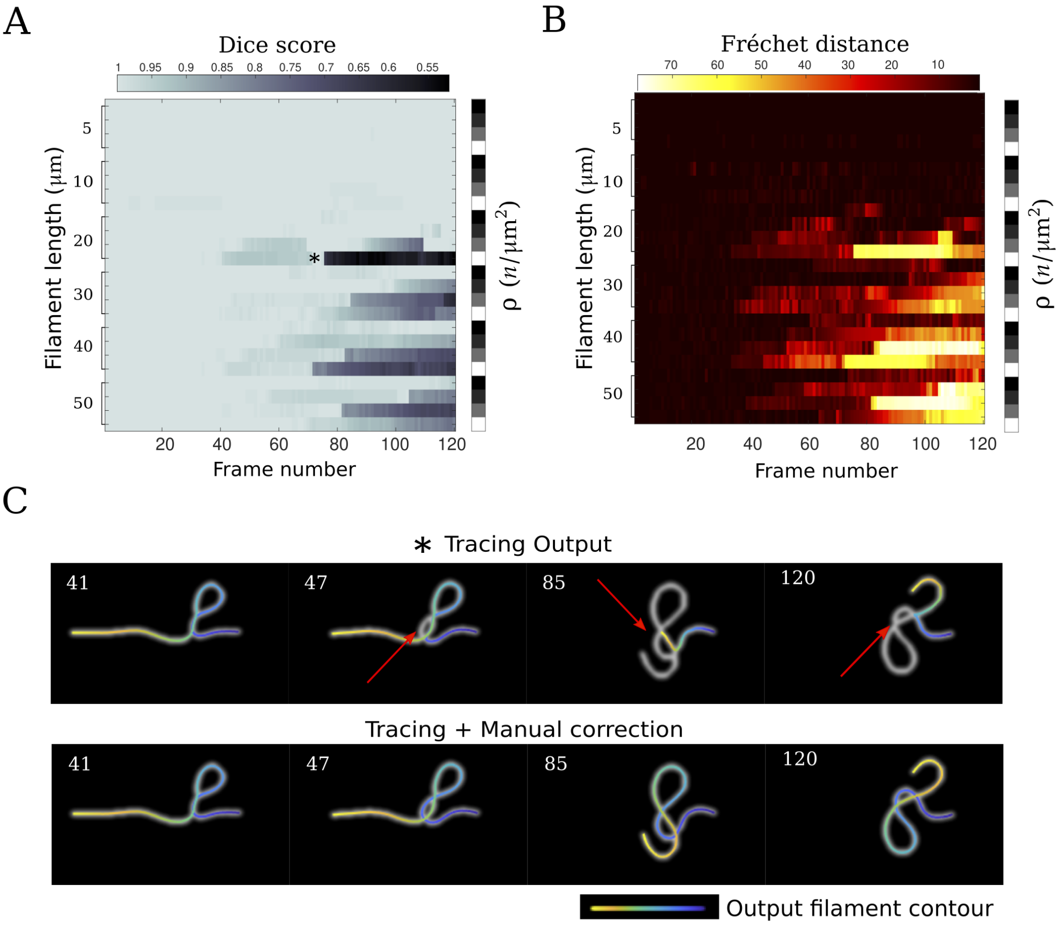

Figure S2. Interactive resolution of complex knots from simulated data. (A) Simulated time-series of filament buckling and bending are used as inputs to KnotResolver. Validity of detected contours are quantified in terms of the Dice score (grayscale colorbar). Simulations were run for increasing MT lengths from 5 to 50 μm (y-axis) and frame-numbers from 1 to 120 (x-axis). Each value of MT length was simulated for four motor densities 12 (black), 25 (dark gray), 50 (light gray) and 100 motors μm^{-2} (white). Asterisks mark the time series selected for further analysis. (B) A similar matrix with the Fréchet distance is plotted. (C) Branch resolution output using manual restarts at problem areas showing correct arrangement of the filament contour.

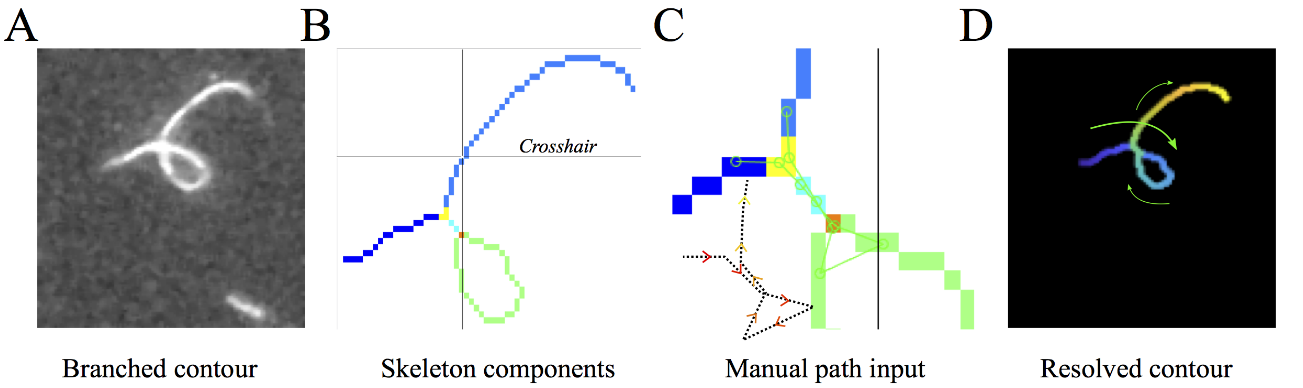

Figure S3. Interactive resolution of complex knots from experimental data. (A) Time-series of a buckling MT filament, with no reference frame that is lacks a knot. (B) The *KnotSegmenter.m* code is used to optimize the segmentation of the knot and the frame number is noted in the *myParams_optimized.csv* under the entry "ManualCheck" with value [p], where p=1 to N (integer values) of the frame number in which the code should allow the user to interactively determine the resolution of the path. When run, the code then pops-up an image of the 'labelled regions', which the user must now connect in sequence (dotted lines with arrows), by clicking on the segments, (C) as they should be correctly resolved (crosshairs). (D) The final segmented output with the input path (arrows) showing the correct arrangement of pixel coordinates along the provided path.

Supplemental videos

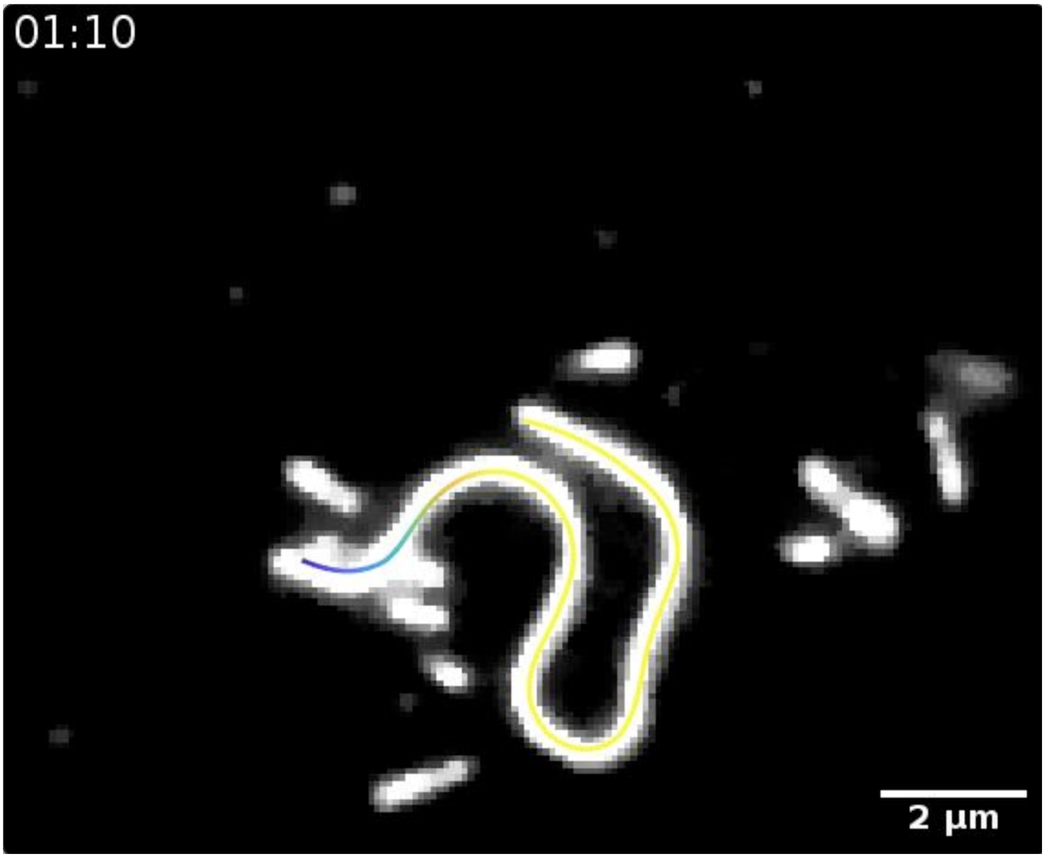

Video SV1. Representative time-series of filament knot resolution with tracked contour. The fluorescence microscopy time-series of a filament undergoing self-intersection (gray) overlaid with the segmented and tracked contours. KnotResolver parameters used to automatically track the series without manual intervention were: intensity threshold = 0.46, contour iteration = 10, contraction bias = 0.4, smooth factor = 0.2. Scalebar: 2 μm, interval between frames (Δt): 10 s.
