# Supplementary figures and images for: KnotResolver: tracking self-intersecting filaments in microscopy using directed graphs
Source: Bioinformatics. 2024 Sep 3;40(9):btae538. doi: 10.1093/bioinformatics/btae538 (PMC11483626; doi:10.1093/bioinformatics/btae538)

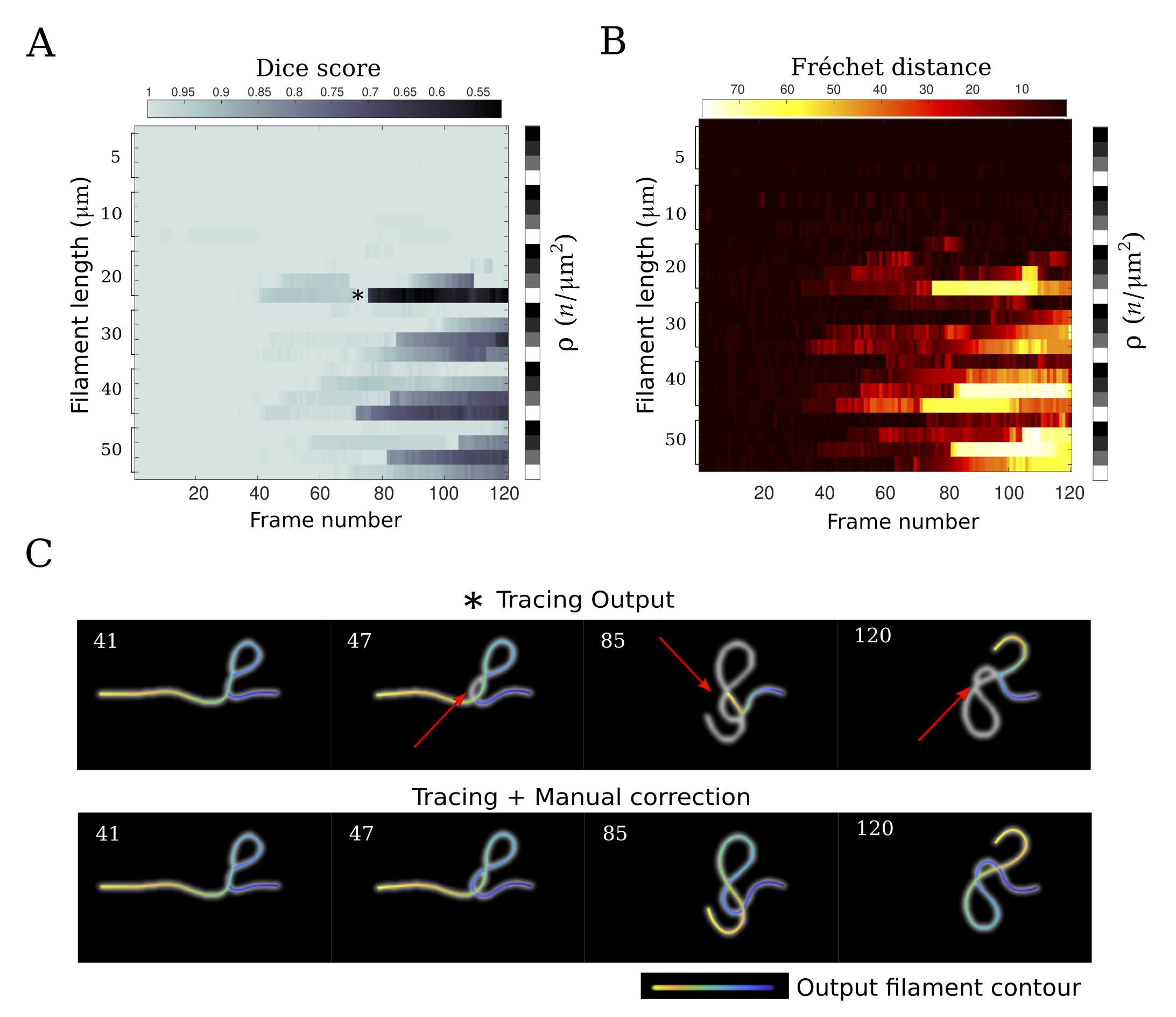

Supplement: btae538_Supplementary_Data [file btae538_Supplementary_Data.zip › FigS2.png]

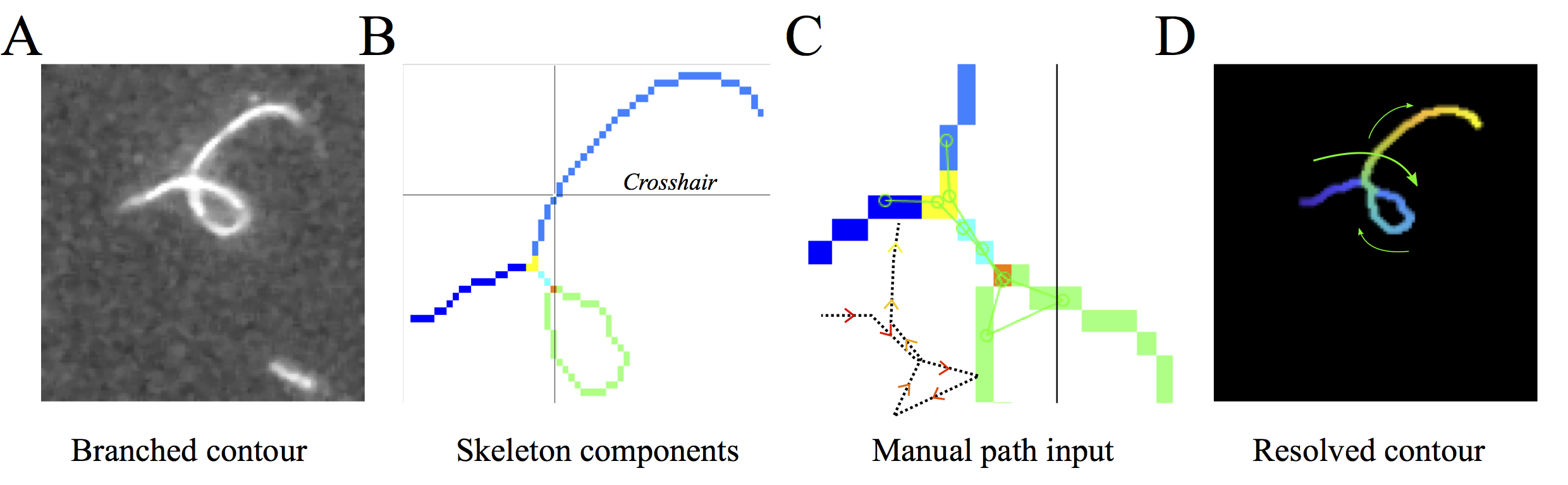

Supplement: btae538_Supplementary_Data [file btae538_Supplementary_Data.zip › FigS3.png]

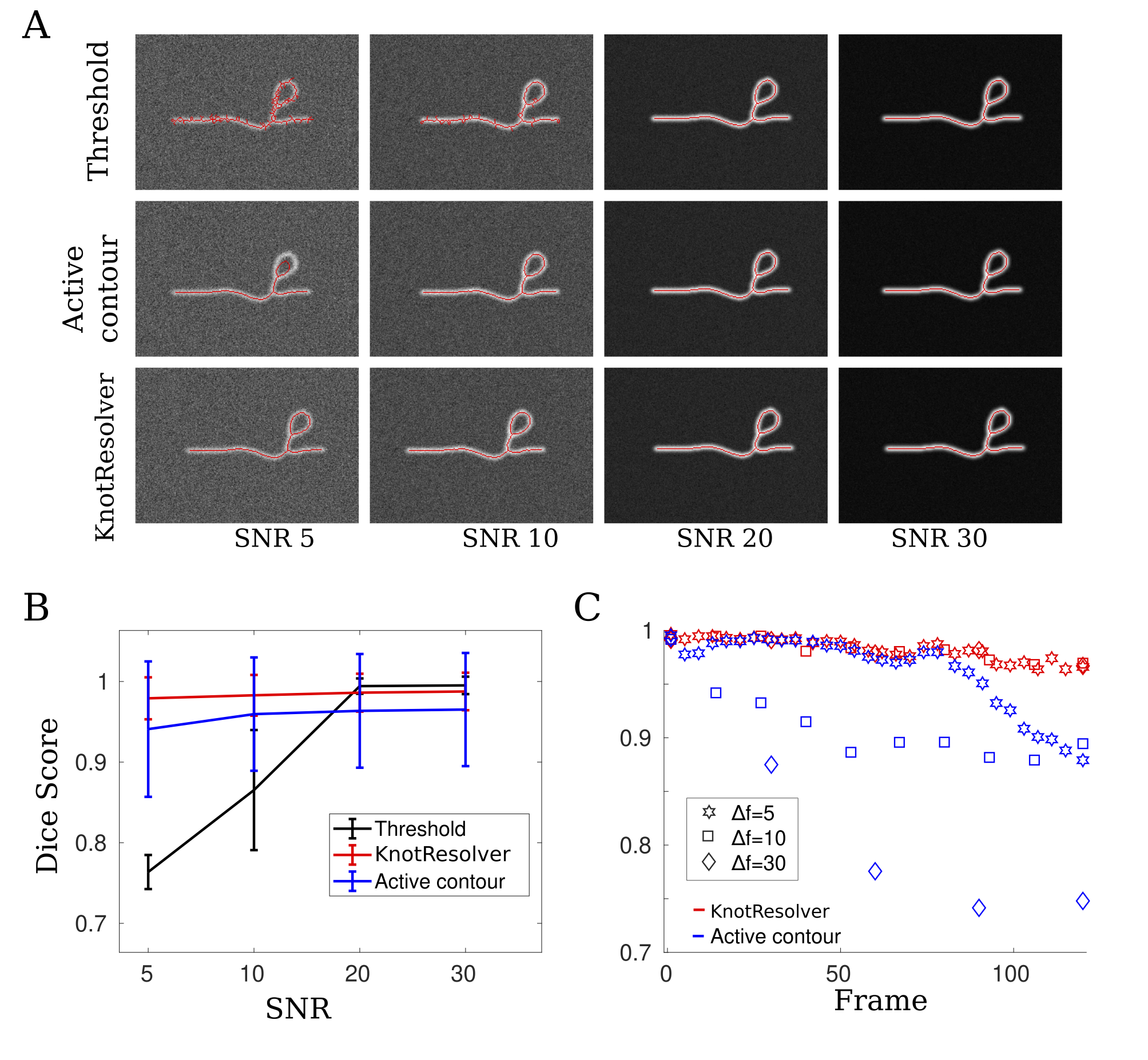

Supplement: btae538_Supplementary_Data [file btae538_Supplementary_Data.zip › FigS1.png]
